# Clinical Diagnosis and Management of Bilateral Plunging Ranula

**DOI:** 10.1155/carm/8833631

**Published:** 2026-04-10

**Authors:** Bo-Wen Liu, Hai-Xiao Zou

**Affiliations:** ^1^ Department of Stomatology, The Second Affiliated Hospital of Nanchang University, Nanchang, China, jxndefy.cn

**Keywords:** imaging, plunging ranula, sublingual gland

## Abstract

Ranula is a benign salivary gland lesion originating from the sublingual salivary gland. This condition is primarily associated with salivary extravasation and retention due to obstruction or injury of the submandibular duct. A plunging ranula represents a rare variant occurring in the neck, submandibular, or submental regions, with bilateral presentations being particularly uncommon. Clinically, due to its lack of specificity, it is easily confused with other cervical lesions. This paper reports a case of a 25‐year‐old female presenting with bilateral painless submandibular masses. MRI revealed the characteristic bilateral “drooping tail sign”. Following bilateral sublingual gland and cyst excision, the patient recovered well postoperatively with no immediate complications. At the two‐month follow‐up, no recurrence was observed, and symptoms were completely resolved. This paper aims to summarize the imaging features of bilateral plunging ranula and enhance clinical capacity for the differential diagnosis of this rare condition.

## 1. Introduction

Ranula is a common adenomatous lesion of the sublingual salivary gland. Based on aetiology, it may be classified as a retention cyst or an extravasation cyst. The former arises from distal obstruction of the submandibular duct, causing proximal dilation due to salivary secretion. The latter, conversely, is associated with salivary leakage into the interstitial space following ductal damage or rupture [1]. Clinically, it manifests as soft, elastic, translucent, pale blue vesicles [2].

Ranula may be classified into simple, plunging, and mixed types based on the location of the cyst. The prevalence of simple ranula is 0.02%, occurring in the sublingual region above the genioglossus muscle [3]. Compared with simple ranulas, ranulas extending beyond the mylohyoid muscle, known as “plunging ranulas,” and those involving both oral and cervical components are extremely uncommon. Plunging Ranula is a circumscribed retention or pseudocyst confined to sublingual location. The mass may penetrate the mylohyoid muscle and extend into the neck, sublingual, or submandibular space. Clinically, they typically present as a nontender lump beneath the jawline [4]. The overall annual incidence rate for plunging ranula is 2.4 cases per 100,000 person‐years. Bilateral involvement is extremely rare in this subtype; in one study of 187 cases of plunging ranula, bilateral lesions accounted for only approximately 9.1% [5]. The mixed type represents a combination of the two aforementioned types, with cysts observable in both the sublingual and submandibular regions [6].

Clinically, rapid diagnosis can be achieved through physical examination; however, definitive classification of ranula relies on imaging studies such as ultrasound, CT, and magnetic resonance imaging (MRI). Due to the lack of specificity of plunging ranula, it is frequently confused with other cystic conditions including dermoid cysts, cystic lymphangiomas, and branchial cleft cysts [7]. This paper aims to summarize the characteristics of bilateral plunging ranula through the presentation of a rare case, examining its anatomical features and imaging findings to aid clinical differential diagnosis.

## 2. Case Presentation

A 25‐year‐old female patient presented at Nanchang County People’s Hospital in September 2025. She reported the spontaneous development of painless masses in both mandibular regions one month prior. These had progressively enlarged to the size of a pigeon’s egg. Intraoral findings including bilateral mild swelling of the floor of the mouth were noted, with slight elevation of the anterior two‐thirds of the tongue; no visible oral ranula (pale blue vesicle) was identified in the sublingual folds, and the orifices of Wharton’s duct were patent and free of salivary fistula or purulent discharge on both sides. Palpation of the extraoral bilateral submandibular masses revealed soft consistency, smooth surfaces, nontenderness, and no adhesion to the overlying skin or underlying deep fascia; the masses were mobile with deglutition and tongue movement. The patient had no history of oral or cervical trauma, surgery, drug allergies, or familial medical conditions related to salivary gland diseases.

**FIGURE 1:**
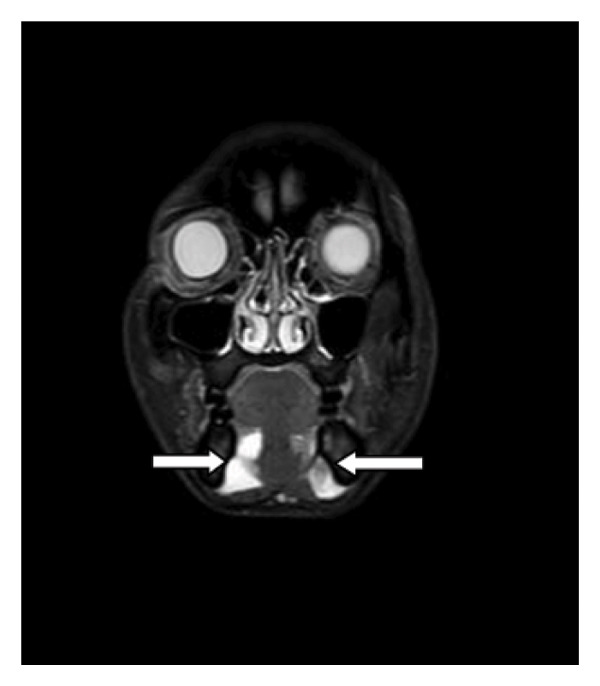
Magnetic resonance imaging of the coronal plane demonstrates bilateral cystic masses adjacent to the mandible, extending into the submandibular space. The masses exhibit well‐defined borders and show no enhancement.

**FIGURE 2:**
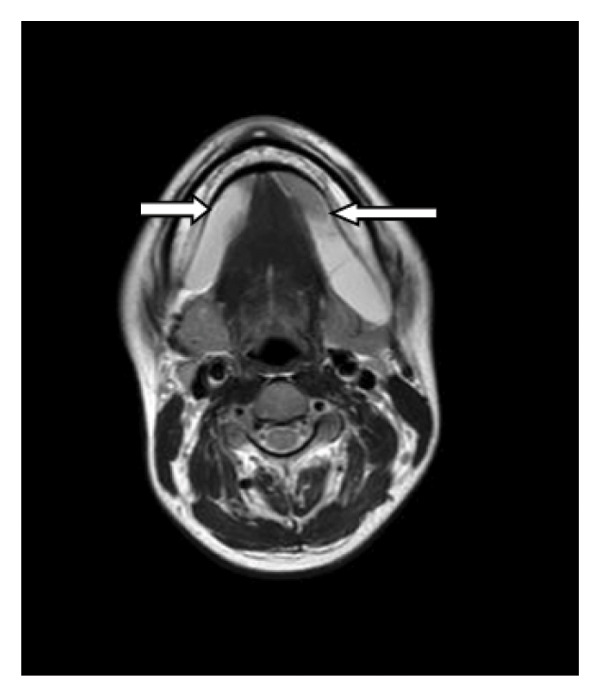
MRI cross‐sectional images reveal bilateral cystic masses in the mandible, extending into the submandibular space. The masses exhibit well‐defined borders and show no enhancement.

**FIGURE 3:**
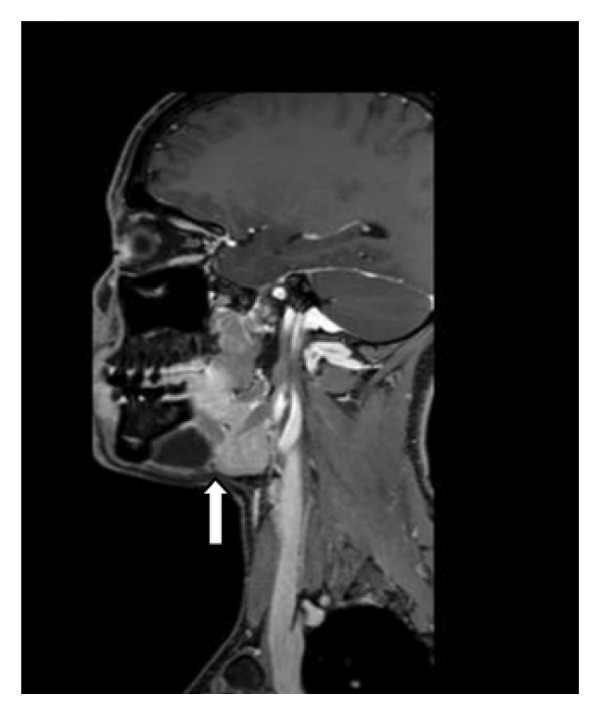
MRI sagittal images reveal bilateral cystic masses in the mandible, extending into the submandibular space. The masses exhibit well‐defined borders and show no enhancement.

The patient underwent MRI. On T1‐weighted imaging (T1WI), bilateral homogeneous hypointense cystic lesions were identified in the submandibular space (inferior to the mylohyoid muscle), adjacent to the bilateral genioglossus muscles and sublingual glands; on T2‐weighted imaging (T2WI), the lesions showed marked homogeneous hyperintensity with well‐defined margins, and the largest lesion (right side) measured approximately 2.6 × 2.3 cm in the axial plane. The classic bilateral “drooping tail sign” was clearly visualized: the main cystic components occupied the bilateral submandibular spaces, with small tail‐like extensions of the cysts passing through the mylohyoid muscle into the bilateral sublingual spaces (superior to the mylohyoid muscle). No enhancement was observed in the cystic lesions or their walls on postcontrast T1WI sequences. Additionally, no narrowing of the oropharynx or larynx was noted, the trachea was uncompressed, and no significant enlargement or abnormal enhancement of cervical lymph nodes was identified (Figures 1, 2, 3). The combined clinical and imaging findings were highly consistent with bilateral plunging ranula.

Following multidisciplinary discussion with the oral and maxillofacial surgery team, bilateral sublingual gland excision + plunging ranula cyst marsupialization and enucleation were planned and performed under general anesthesia via a pure intraoral surgical approach (no extraoral incision). A curved incision was made along the bilateral sublingual folds (mucobuccal fold) in the floor of the mouth; the oral mucosa was elevated to expose the bilateral sublingual glands and the cystic lesions extending to the submandibular space through the mylohyoid muscle defect. The sublingual glands were carefully dissected and completely excised bilaterally to eliminate the source of salivary secretion. The cystic sacs were enucleated as completely as possible, with residual cyst walls marsupialized to the oral mucosa to prevent reaccumulation of saliva. Wharton’s duct was carefully identified and preserved bilaterally throughout the procedure to maintain submandibular salivary gland function; no injury to the duct or adjacent lingual nerve was noted. Two small Penrose drains were placed in the bilateral submandibular spaces via the intraoral incisions, with the drain ends externalized through the floor of the mouth mucosa. The intraoral incisions were closed with absorbable sutures (4‐0 Vicryl). No recurrence of the bilateral plunging ranula was observed at the 2‐month follow‐up, and all clinical symptoms were completely resolved.

## 3. Discussion

Ranula is a common benign cystic lesion caused by obstruction or damage to the sublingual gland. It accounts for 6% of oral salivary gland cysts, with a male‐to‐female ratio of approximately 1:1.3 [8]. Plunging ranula typically presents as a mass in the mandibular region, covered by only a thin layer of mucosa. On palpation, it is soft and nonadherent to the skin, commonly occurring in the neck, submandibular, or submental areas [9]. Bilateral plunging ranula arising simultaneously from ductal structural abnormalities or glandular injury in both sublingual glands is exceptionally rare in clinical practice. Consequently, establishing the imaging characteristics of bilateral plunging ranula is crucial for differentiating it from cystic conditions such as dermoid cysts, cystic lymphangiomas, and branchial cleft cysts.

Plunging ranula typically presents on MRI or CT imaging as a well‐defined, solitary lesion with no calcifications and a water‐like signal without enhancement. The “drooping tail sign” constitutes its characteristic radiological feature, wherein the cyst primarily occupies the submandibular space, with the remainder extending into the sublingual space posterior to the mylohyoid muscle [10]. Both lesions in this patient exhibited the characteristic “drooping tail sign” with a water‐like signal, well‐defined margins, and no enhancement. Currently, there are very few reported cases demonstrating this typical imaging feature bilaterally concurrently.

Differential diagnosis from other cystic conditions: (1) Simple ranula is typically confined to the sublingual region above the mylohyoid muscle, often presenting with a fluctuant sensation on palpation [6]. (2) Second branchial cleft cysts are commonly located in the lateral aspect of the carotid space, anterior, and medial to the sternocleidomastoid muscle. On CT and MRI, they appear as well‐defined, low‐density shadows. The surrounding cyst wall is smooth and thickened, demonstrating enhancement on contrast‐enhanced imaging [11]. (3) Dermoid cysts are commonly found in the floor of the mouth and submental region. CT and MRI scans reveal mixed density signals, with floating bodies within the cyst cavity exhibiting the “marble sign”. Calcification may occur within these cystic bodies [12]. (4) Cystic lymphangioma is most commonly found in the neck and supraclavicular regions, though it may also occur in the submandibular area. The lesion presents with surface distension and yields a positive transillumination test. Imaging demonstrates a multichambered cystic low‐density lesion, with visible fluid‐fluid levels within the cysts [13].

Therefore, when imaging studies reveal bilateral cystic lesions in the submandibular region, the possibility of bilateral plunging ranula should be considered. Confirmation of the “drooping tail sign” is key to its radiological diagnosis. In addition, ultrasound is an excellent technique for the diagnosis of both ranulas and plunging ranulas [14]. Ultrasound not only demonstrates the characteristic defects of the mylohyoid muscle, sublingual gland herniation, and involvement of the cervical spaces characteristic of plunging ranula but also allows real‐time observation of the demonstration process of sublingual salivary gland herniation. Since the patient did not undergo ultrasound examination, ultrasound images could not be provided in the present case, which constitutes the limitation. Therapeutically, simultaneous excision of both sublingual glands and cysts represents the gold standard for radical cure of bilateral lesions and prevention of recurrence. Sclerotherapy has been attempted for simple ranula, but has shown limited efficacy for plunging ranula [15].

## 4. Conclusions

This rare case report describes a 25‐year‐old female patient with bilateral plunging ranula, who had no other significant medical history. Differential diagnoses included simple ranula, second branchial cleft cyst, dermoid cyst, and cystic lymphangioma. This case study aims to emphasize that bilateral painless neck masses should prompt consideration of such rare pathologies. Detailed imaging evaluation—particularly CT and MRI—is crucial for establishing a definitive diagnosis and formulating an appropriate surgical strategy.

## Funding

No funding was received for this manuscript.

## Consent

Written informed consent was obtained from the patient for publication of this case report.

## Conflicts of Interest

The authors declare no conflicts of interest.

## Data Availability

The data that support the findings of this study are available on request from the corresponding author. The data are not publicly available due to privacy or ethical restrictions.
